# A Physics-Informed Neural Network Approach for Nearfield Acoustic Holography

**DOI:** 10.3390/s21237834

**Published:** 2021-11-25

**Authors:** Marco Olivieri, Mirco Pezzoli, Fabio Antonacci, Augusto Sarti

**Affiliations:** Dipartimento di Elettronica, Informazione e Bioingegneria (DEIB), Politecnico di Milano, Piazza Leonardo da Vinci 32, 20133 Milan, Italy; mirco.pezzoli@polimi.it (M.P.); fabio.antonacci@polimi.it (F.A.); augusto.sarti@polimi.it (A.S.)

**Keywords:** nearfield acoustic holography, convolutional neural network, Kirchhoff–Helmholtz integral, finite element method

## Abstract

In this manuscript, we describe a novel methodology for nearfield acoustic holography (NAH). The proposed technique is based on convolutional neural networks, with autoencoder architecture, to reconstruct the pressure and velocity fields on the surface of the vibrating structure using the sampled pressure soundfield on the holographic plane as input. The loss function used for training the network is based on a combination of two components. The first component is the error in the reconstructed velocity. The second component is the error between the sound pressure on the holographic plane and its estimate obtained from forward propagating the pressure and velocity fields on the structure through the Kirchhoff–Helmholtz integral; thus, bringing some knowledge about the physics of the process under study into the estimation algorithm. Due to the explicit presence of the Kirchhoff–Helmholtz integral in the loss function, we name the proposed technique the Kirchhoff–Helmholtz-based convolutional neural network, KHCNN. KHCNN has been tested on two large datasets of rectangular plates and violin shells. Results show that it attains very good accuracy, with a gain in the NMSE of the estimated velocity field that can top 10 dB, with respect to state-of-the-art techniques. The same trend is observed if the normalized cross correlation is used as a metric.

## 1. Introduction

Nearfield acoustic holography (NAH) [1,2] is an interesting acoustic-based technique for the contactless analysis of vibrating structures, such as plates and shells. NAH represents an appealing alternative to vibrational analysis carried out with accelerometric sensors when, for example, the structure under analysis is particularly fragile or the deployment of accelerometers is not feasible. Contactless analysis is also preferred when lightweight objects are considered, since no additional mass needs to be added. Differently from contactless optical techniques, e.g., laser Doppler vibrometer (LDV), NAH can be employed with objects made of reflective materials.

NAH estimates the velocity field of a vibrating structure starting from acoustic measurements acquired in its proximity. The sound pressure is typically captured by a microphone array deployed on a plane, known as holographic plane. The holographic plane is close to the vibrating surface in order to measure the evanescent waves, which are confined in the proximity of the structure [2]. With the aim of estimating the velocity field of the source from the pressure on the holographic plane, NAH relies on the inversion of the well-known Kirchhoff−Helmholtz (KH) integral [2,3]. As a matter of fact, the KH equation relates the normal velocity of a vibrating surface to the acoustic pressure generated by the vibration. Sometimes NAH is also cast as a sound field reconstruction problem [4] for applications in the field of source characterization [5] and sound field navigation [6,7,8]. The inversion of the KH integral, targeted by NAH, is known to be a highly ill-conditioned problem. Hence, many regularization strategies for NAH have been proposed in the literature [9,10,11].

The KH integral can be numerically computed through the boundary element analysis (BEA) [12,13]. Therefore, a possible solution to NAH is implemented through the inversion of BEA (IBEA) [14,15], using Tikhonov regularization. This technique is able to provide accurate results, but its application is limited by the high computational cost.

An alternative regularization strategy is represented by compressed sensing (CS) in [16,17]. One of the former approaches in this direction was proposed in [16] and named as Nearfield ACoustic HOlography with Sparse regularization (NACHOS). It aims at approximating the vibrational data through a linear combination of a limited number of plane-wave basis functions. However, its use is limited to star-shaped planar plates.

A different approach to NAH is given by the equivalent source method (ESM) [18,19]. This model assumes that the measured acoustic pressure field radiated by the source can be equivalently expressed as the soundfield generated by a set of point-like virtual sources located within or in proximity of the real source. The NAH techniques based on ESM are typically structured in two steps. First, the weights of the equivalent sources are estimated by minimizing the error between the measurements and the soundfield obtained by propagating the equivalent sources. Successively, the target velocity field is determined by propagating the equivalent sources to the surface of the vibrating structure. An aspect that greatly impacts on the accuracy of ESM is the choice of number and location of th equivalent sources. To the authors knowledge, only rules of thumb are proposed, which are not, however, applicable in some contexts. In order to deal with this problem, ESM techniques based on CS [20,21,22] have been proposed with the aim of finding small and sparse subsets of equivalent sources.

For example, Canclini et al. [22] proposed building a dictionary of equivalent sources in order to solve NAH. This technique, called dictionary-based esm (DESM), exploits synthetic data for finding the equivalent sources. The resulting set is compressed using principal component analysis and then it constitutes the learned dictionary. However, the dictionary is specialized for a single rectangular object with fixed dimensions and validated on similar objects with different mechanical parameters.

Another method that proves the importance of having a non-redundant representation of the observed data is presented by Fernandez-Grande et al. in [20]. Authors proposed a compressive-ESM (CESM) solution for NAH obtaining a sparse representation of the measured wave field. In particular, they can find the equivalent source weights through the computation of a $\ell_{1}$-norm minimization problem. Nevertheless, although CESM can be applied to different vibrating objects with different dimensions and geometries, determining the location and the number of equivalent sources is still an open problem.

Recently, a new approach to NAH based on deep learning [23] has been proposed in [24]. The authors, inspired by the effectiveness of learned features for NAH [22] and the well-known feature learning capabilities of deep neural networks (DNN) in the context of acoustics [25,26,27,28,29], proposed a convolutional neural network (CNN) [30] for performing NAH. The promising approach of [24] provides accurate results, but the evaluation is limited to rectangular plates of isotropic material only. Moreover, it considers a dense spatial sampling of the hologram at a minimal distance from the source, which limits the adoption to laboratory measurements.

In order to overcome these limitations, in [31] the authors proposed an enhanced version of [24]. The architecture in [31], called super resolution CNN (RCNN), is able to perform NAH with 3D structures of complex shape and arching profiles, also built from orthotropic material as violin top plates. In addition, while retaining the same output resolution obtained in [24], the number of required pressure points, namely the number of microphones in the array, in [31] is reduced, achieving super resolution of the data. Although the SRCNN-based NAH approach is able to estimate the velocity magnitude field on different shapes of violin top plates, it can estimate only the magnitude of the vibrational field discarding the phase information. Moreover, the solution in [31] has no prior information about the physical problem. Indeed, SRCNN acts an image mapping between the input and output learned from the specific training set without considering any physical information.

In this manuscript, we propose a novel approach to solve the NAH problem. The goal of this work is twofold. On the one side, we combined the advantages of deep learning solutions, in particular CNNs, with prior knowledge coming from the physical model, which leads NAH, i.e., the KH integral. On the other side, we built an architecture able to estimate both the magnitude and the phase information of the normal velocity field on the vibrating surface.

The proposed model consists of two main blocks. The former takes the form of a CNN with one input, the hologram pressure field, and two outputs, i.e., the pressure and velocity fields on the vibrating surface. Successively, the second block propagates the two network outputs with the Kirchhoff−Helmholtz model in order to provide an estimate of the pressure at the hologram. For this reason, we called the devised architecture Kirchhoff−Helmholtz-based CNN (KHCNN).

We focused on the velocity analysis on rectangular plates and violin top plates starting from a corrupted version of acoustic pressures at the hologram plane with additive noise. Moreover, in order to consider scenarios compatible with experimental measurements, we performed NAH starting from a low number of pressure points at the hologram, namely the number of microphone sensors.

The proposed method is validated comparing the predicted vibrational fields with the ground truth and estimates given by CESM [20]. Moreover, we compared the performances also with respect to the fully data-driven approach of SRCNN-based NAH presented in [31]. Simulation results confirm the effectiveness of the proposed KHCNN approach to NAH. In particular, the presence of the physical model block improves the velocity accuracy of the network estimates.

It is worth noticing that the dataset that we proposed does not present a Gaussian distribution of the data [32]. Interestingly, the devised approach is able to accurate model this variability of the dataset.

The paper is structured as follows. Section 2 presents the data model of the problems. In Section 3, the mathematical formulation adopted and the overall methodology is introduced. The description of the proposed KHCNN along with the training procedure are reported in Section 4. Section 5 presents the generation of the simulated datasets. The validation results with the comparison between the state-of-the-art approaches are report in Section 6. A discussion of the available experiments is present in Section 7. Finally, Section 8 draws some final conclusions.

## 2. Data Model

### 2.1. Data Model of the Mechanical Vibration

The characterization of a vibrating structure requires the knowledge of its structural dynamic properties. An essential information is represented by the modes of vibration. Modes, also called eigenmodes, are natural patterns of deformation that occur in objects during vibrations. They are associated to the modal frequencies or eigenfrequencies. Indeed, at these specific frequencies, the structural vibrations produce a stationary wave, the so-called mode shape. These vibrational patterns are characterized by nodes and anti-nodes. The former are points where no displacement of the structure is observed, whereas in the latter, maximum deformation occurs.

It is worth noticing that modes are inherent properties of a structure; thus, they do not depend on external forces or loads acting on the structure. Modes depend only on the object geometry, the material properties (i.e., mass, stiffness, damping properties) and from the boundary conditions (BCs) applied to the structure (i.e., simply supported, free or clamped BCs) [33].

Conversely, when a structure is excited by external forces, its vibration results in the Operational Deflection Shape (ODS) [34]. In particular, the ODS represents a combination of modes giving a general description for the harmonic evolution of the displacement over surface. Unlike mode shapes, the ODS depends on the excitation point, the load applied to the structure, and the frequency content of the excitation signal [34].

### 2.2. Data Model of the Acoustic Behavior

The pressure radiation produced by the points s belonging to a vibrating surface S and measured in a point r is predicted by the Kirchhoff−Helmholtz (KH) integral [2], i.e.

(1) $$\alpha \left(r\right)p\left(r,\omega \right)=\int_{S}\left(p\left(s,\omega \right)\frac{\partial }{\partial n}g_{\omega }\left(r,s\right)-g_{\omega }\left(r,s\right)\frac{\partial }{\partial n}p\left(s,\omega \right)\right)dS,$$

where ω is the angular frequency of vibration, p(·,ω) is the pressure field, n is outward normal vector and $g_{\omega }\left(r,s\right)$ is the free-field Green’s function from s to r, namely

(2) $$g_{\omega }\left(r,s\right)=\frac{1}{4\pi }\frac{e^{-j\frac{\omega }{c}\|r-s\|}}{\left\|r-s\right\|},$$

with *c* the sound speed in the air and *j* is the imaginary unit. Notice that the computation of (1) depends on the parameter α(r), which is determined by the position of the radiation point r:

(3) $$\alpha \left(r\right)=\left\{\begin{array}{cc} 1, & \mathrm{if}\;r\;\mathrm{is}\;\mathrm{outside}\;S\\ 1/2, & \mathrm{if}\;r\;\mathrm{is}\;\mathrm{on}\;S\\ 0, & \mathrm{if}\;r\;\mathrm{is}\;\mathrm{inside}\;S \end{array}\right..$$

Moreover, (1) has to satisfy the Sommerfeld condition, which gives a boundary condition at infinity [2,35], namely

(4) $$\lim_{r\to \infty }r\left[\frac{\partial }{\partial n}p\left(r\right)-jkp\left(r\right)\right]=0.$$

The Euler’s equation [2] defines a fundamental relation between the pressure and the normal velocity and writes

(5) $$\frac{\partial }{\partial n}p\left(s,\omega \right)=j\omega \rho_{0}v_{n}\left(s,\omega \right),$$

where $\rho_{0}$ is the mass density of the material, which for the air medium at $20\;{}^{\circ }C$ is $\rho_{0}\approx 1.225\;kg\;\cdot \;m^{-3}$ and $v_{n}\left(s,\omega \right)$ is the normal velocity in point s. By substituting (5) in (1), we can derive a different formulation of the KH integral equation for the exterior radiation problem of a vibrating structure

(6) $$p\left(r,\omega \right)=\int_{S}\left(p\left(s,\omega \right)\frac{\partial }{\partial n}g_{\omega }\left(r,s\right)-j\omega \rho_{0}v_{n}\left(s,\omega \right)g_{\omega }\left(r,s\right)\right)dS.$$

Thanks to this formulation of the KH integral, we can compute the pressure radiated by a vibrating source starting from the knowledge of the pressure and the normal velocity fields on the object’s surface.

### 2.3. Notation in Nearfield Acoustic Holography

**Figure 1:**
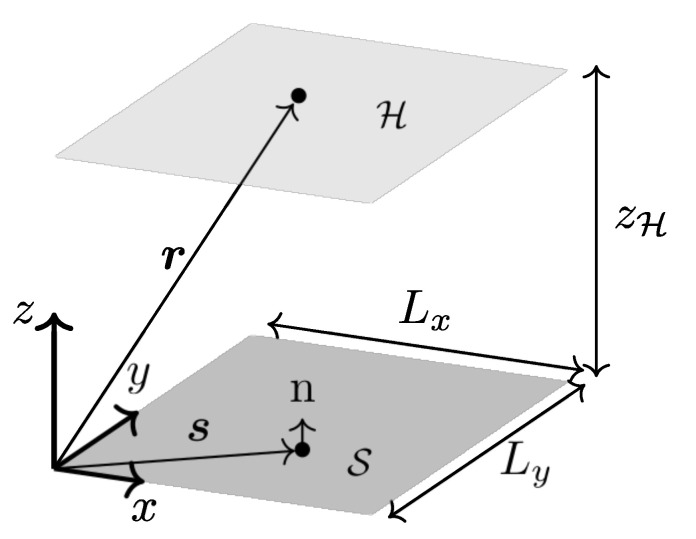
General setup for NAH. The vibrating surface S is a finite plane, vectors r, s and n are defined according to a Cartesian reference system located at the bottom-left corner of S. The radiated soundfield is acquired by a microphone array placed in the hologram plane H at elevation $z_{H}$.

In NAH, the soundfield on the holographic plane H is acquired through a microphone array nearby the object. In a Cartesian coordinate system, a general setup for performing NAH is shown in Figure 1, where H is horizontal and its *z* coordinate is $z_{H}$.

In solid media, the vibrating structure radiates sound at frequencies higher than the cutoff frequency [2]. At frequencies lower than the cutoff, the soundfield decays exponentially with *z*, generating the evanescent waves. For this reason, the near-field condition [2] is an essential requirement in NAH for capturing all the velocity field components.

In the NAH context, we are interested in solving the inverse propagation problem. In practice, this boils down to the inversion of (6), i.e.,

(7) $$v_{n}\left(s,\omega \right){|}_{\begin{array}{c} s\in S \end{array}}\approx F^{-1}\left(p\left(r,\omega \right)\right){|}_{\begin{array}{c} r\in H \end{array}},$$

where F is a discrete estimator that approximates the soundfield on the hologram plane. However, the inverse propagation problem (7) is highly ill-conditioned, thus requiring a regularization procedure.

In this work, $F^{-1}$ takes the form of a CNN. From the input pressure field at the hologram, the devised network is able to estimate the velocity field on the vibrating surface, thus avoiding explicit matrix inversions.

## 3. Problem Formulation

In this manuscript, we present a novel approach to NAH that combines the advantages of deep learning [23] with the physical model of acoustic propagation (6). The underlying physical model allows us to enrich the recent data-driven NAH approaches in [24,31] with an estimate of the complex velocity field on the object’s surface (i.e., both magnitude and phase information) to better characterize the vibrational behavior of the source.

Let us now consider a sampled version of the radiated pressure field acquired through a uniform planar microphone array placed on the horizontal holographic plane H. Hence, the hologram pressure field in matrix form is

(8) $$P_{H}\left(\omega \right)\in C^{M_{1}\times M_{2}},$$

where the microphones are located at $r_{m_{1}m_{2}}$ with $m_{1}=1,\cdots ,M_{1}$ and $m_{2}=1,\cdots ,M_{2}$. $M_{1}$ and $M_{2}$ are the number of points in the array along the *y* and *x* axes, respectively.

Similarly, we can define a sampled version of the normal velocity field and of the pressure field on the object’s surface in matrix form as

(9) $$V\left(\omega \right)=v_{n}\left(s_{n_{1}n_{2}},\omega \right){|}_{\begin{array}{c} s_{n_{1}n_{2}}\in M \end{array}}⊙B\in C^{N_{1}\times N_{2}},$$

(10) $$P_{S}\left(\omega \right)=p\left(s_{n_{1}n_{2}},\omega \right){|}_{\begin{array}{c} s_{n_{1}n_{2}}\in M \end{array}}⊙B\in C^{N_{1}\times N_{2}},$$

where ⊙ is the Hadamard product, M is a rectangular mesh grid on the source samples at $s_{n_{1}n_{2}}$ with $n_{1}=1,\cdots ,N_{1}$ and $n_{2}=1,\cdots ,N_{2}$, such that it entirely contains the geometry of the vibrating surface S. In order to take into account the shape of the object, let B be a binary mask, which selects the points of the mesh grid belonging to the target surface. In particular, $(b_{n_{1}n_{2}})=1$ if $s_{n_{1},n_{2}}$ lies on the surface, and 0 otherwise.

With the above definitions at hand, we can write the discretized Kirchhoff−Helmholtz Equation (6) in matrix form as

(11) $$p_{H}\left(\omega \right)\approx F\left(p_{S},v,\omega \right)=G_{p}^{H}\left(\omega \right)p_{S}\left(\omega \right)-j\omega \rho_{0}G_{v}^{H}\left(\omega \right)v\left(\omega \right),$$

where ${}^{H}$ represents the Hermitian transpose operator, $p_{H}\in C^{M\times 1}$, and $p_{S}$, $v\in C^{N\times 1}$ are the column vector forms of $P_{H}$, $P_{S}$ and V, respectively. Likewise, $G_{v}\in C^{N\times M}$ is the matrix of Green’s functions relating the *N* points on the surface with the *M* points on the hologram and $G_{p}=\frac{\partial }{\partial n}G_{v}$.

Notice that in (11), the number of points *M* can be different from *N*. In typical NAH experimental scenarios M<N, thus having a limited number of microphone sensors available with respect to the desired velocity resolution. Moreover, the discrete estimator F represents an estimate of the real pressure field, with accuracy determined by the number of adopted discrete points.

**Figure 2:**
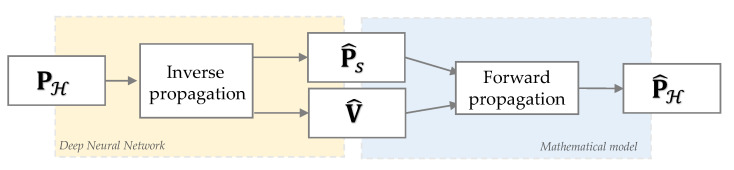
Scheme of the overall two-block scheme proposed to solve the NAH problem. The outputs of the DNN (yellow block) are fed to the forward propagation mathematical model (blue block) in order to get an estimate of the hologram pressure.

Figure 2 shows the two-block approach proposed in this paper to combine data-driven and model-based solutions of the NAH problem.

Inspiring by the recent works of CNN–NAH architectures presented in [24,31], where deep learning solutions have proved the ability to extract a powerful feature representation to regularize the inverse NAH problem, we employed a DNN to infer the back propagation relation. In particular, from the input pressure field acquired at the hologram plane $P_{H}\left(\omega \right)$, the first block reconstructs the complex fields $\hat{V}\left(\omega \right)$ and ${\hat{P}}_{S}\left(\omega \right)$ on the object’s surface.

On the other hand, the use of physical information makes prior knowledge useful to regularize the ill-posed inverse problem, as shown for example in ESM-based NAH techniques. For this reason, we fed a second block with the two DNN outputs in order to apply a mathematical model of the forward acoustic propagation. This implies also knowing the Green functions relating the reconstruction points in S with the measurements locations on H and the frequency at which the object is vibrating. Thanks to this second block, we can obtain the estimate of ${\hat{P}}_{H}\left(\omega \right)$, comparing it with the input pressure, and tuning the performance of the DNN.

## 4. Network Description

In this section, we describe in detail the model sketched in Figure 2 along with the definition of the input and output data and the DNN architecture.

### 4.1. Kirchhoff–Helmholtz-Based Convolutional Neural Network

The estimated pressure field ${\hat{P}}_{H}\left(\omega \right)$ is computed using the discretized version of the KH equation defined in (11). In addition to $P_{S}\left(\omega \right)$ and V(ω), this mathematical model requires knowing the Green function (2) between s and r for all the surface and hologram grid points pairs.

The DNN model adopted to solve the back propagation problem is inspired by the architecture of the renowned U-Net [36]. This architecture consists of three main components: the contraction, the bottleneck, and the expansion sections. Nevertheless, we modified such architecture in order to have two different outputs from the CNN, i.e., the pressure ${\hat{P}}_{S}\left(\omega \right)$ and the velocity $\hat{V}\left(\omega \right)$. Therefore, the proposed model consists of one encoder E and two decoders $D_{1}$ and $D_{2}$.

In order to apply the KH propagation model from the network outputs, the CNN has to reconstruct both output fields in complex domain. Therefore, the input and output data of the devised network are arranged in tensors with two channels containing the real and imaginary parts of the complex fields, respectively, thus preserving magnitude and phase information.

**Figure 3:**
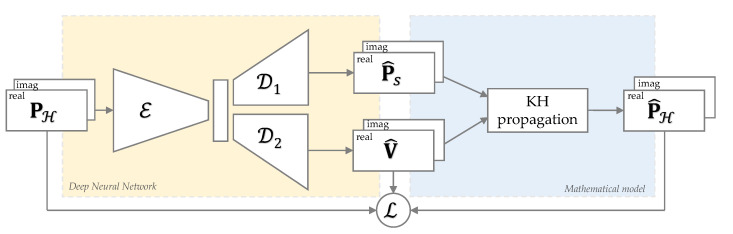
Overall scheme of the proposed KHCNN model. The CNN architecture (yellow block) predicts the real and imaginary parts of ${\hat{P}}_{S}$ and $\hat{V}$ (stacked in two channels) from the input $P_{H}$. The two outputs are then propagated with the KH model in order to obtain the estimate of ${\hat{P}}_{H}$. A proper loss function is built on top of the velocity ground truth and the pressure at the hologram.

For these reasons, we refer to the devised model as Kirchhoff−Helmholtz-based convolutional neural network (KHCNN) and the overall scheme is depicted in Figure 3.

Notice that in Figure 3, real and imaginary parts are stacked to emphasize the fact that they are arranged in two channels. This way, real and imaginary parts are not treated as separate signals, but a feature sharing between the real and imaginary parts during the training process of the network is achieved.

### 4.2. Input/Output Data

The network input is the pressure field acquired at the hologram plane $P_{H}\left(\omega \right)$ arranged in a tensor of $M_{1}\times M_{2}\times 2$. In particular, $M=M_{1}\times M_{2}$ is the number of points used to sample the hologram pressure and the last two channels contain the real and imaginary part of the complex field.

On the other hand, the two outputs of the CNN coming for decoders $D_{1}$ and $D_{2}$ are the pressure ${\hat{P}}_{S}\left(\omega \right)$ and the velocity $\hat{V}\left(\omega \right)$ on the vibrating surface, respectively. Both outputs are arranged in a tensor with dimensions $N_{1}\times N_{2}\times 2$ in order to estimate the real and imaginary parts of the complex fields in the $N=N_{1}\times N_{2}$ points on the object’s surface.

It is worth noticing that the value ranges of $P_{H}\left(\omega \right)$ and V(ω) are different. This is due not only to the different physical quantities and the elevation of the hologram pressure, but it also depends on the geometry and boundary conditions of the vibrating source. Therefore, the input pressure and the output velocity datasets have been normalized with respect to their maximum absolute value; thus, collecting images with magnitude in [0,1]. Notice that this operation does not affect the phase information, which remains unaltered, but only the real and imaginary part of the complex fields, which now are in [−1,1].

Differently from [24], where the pressure on the surface $P_{S}\left(\omega \right)$ was considered as an implicit latent variable, here we consider it as explicit latent variable. Hence, we let KHCNN estimate ${\hat{P}}_{S}\left(\omega \right)$ from the evaluation of the KH propagation model on ${\hat{P}}_{H}\left(\omega \right)$.

### 4.3. CNN Structure

Here we describe the layers of the CNN architecture. We decide to compare two different networks that differ from the input dimensions and the number of parameters.

The first CNN aims to estimate V(ω) (9) starting from the input pressure $P_{H}\left(\omega \right)$ (8), having the same spatial resolution of the output, i.e., same dimension of $M_{1}\times M_{2}=N_{1}\times N_{2}=16\times 64$. This architecture is similar to the one proposed in [24] and it is used here only as a benchmark, since in practical NAH scenarios it is infeasible to measure the hologram pressure in 1024 points due to wiring and spacing problem. For this reason, inspired by the SRCNN approach in [31], we considered another architecture that produces the velocity estimate in 1024 points, but starting from 64 points at the input pressure acquired in a grid with dimension 8×8.

For the sake of simplicity, here we describe only the CNN architecture with M=64 points at the input. Indeed, the benchmark model with the input pressure in M=1024 points presents the same architecture with only an adaptation on the dimensionality.

The proposed encoder E consists of a series of four downsampling blocks. Each block includes two consecutive layers of 2D convolutions with filter size 3×3 and with a rectified linear unit (ReLU) activation function [37]. Moreover, batch normalization and 2×2 max pooling operations are applied after each downsampling block.

From the bottleneck embedding, the expansion section is achieved by two parallel decoders, $D_{1}$ and $D_{2}$, with the same structure. Each upsampling step of both decoders consists of a Conv2DTranspose layer [38] with stride 2×2 followed by two convolutions with ReLU functions and batch normalization. Moreover, skip connections [39] between each downsampling block of the encoder E and the corresponding upsampling layers of $D_{1}$ and $D_{2}$ are used to enable the reuse of the encoded features. The desired output dimensions is reached with a super resolution section consisting in two additional upsampling blocks with asymmetric strides 1×2 and a final layer with linear activation function.

As a consequence, we obtain a double symmetric structure with one shared encoder and two parallel decoders.

### 4.4. Training Procedure

The CNN model is built to extract an estimate of the velocity V(ω) and pressure $P_{S}\left(\omega \right)$ on the vibrating surface starting from the input pressure $P_{H}\left(\omega \right)$. Moreover, the KH model computes ${\hat{P}}_{H}\left(\omega \right)$ from the network outputs. Notice that the quality of the network estimate can be assessed by the accuracy of both the soundfield on the hologram (input of the network) and of the velocity field (output of the network), since the former is estimated through the KH discretized operator from the latter.

Therefore, we define the following mean square error (MSE) loss function:

(12) L=12‖Rev−Rev^‖22+12‖RepH−Rep^H‖22+12‖Imv−Imv^‖22+12‖ImpH−Imp^H‖22,

where Re(·) and Im(·) are operators that take the real and imaginary part of the complex field, respectively. Moreover, the pressure and velocity fields in (12) are represented as column vectors and without the dependence of ω for the sake of simplicity.

The network is implemented (https://github.com/polimi-ispl/nah-khcnn, accessed on 20 November 2021) in Python using Keras [40] and trained through Adam optimizer with the default parameters presented in [41]. We decreased the learning rate by a factor of 0.2 on learning plateau. Moreover, we applied the early stopping regularization technique to prevent overfitting. Hence, we stopped the training after 20 epochs in which no improvement of the validation loss was observed.

## 5. Dataset Generation

We evaluated the proposed approach using two different vibrating structures: aluminum rectangular plates and violin top plates made of Sitka spruce [42]. In the former case, the vibrating object are planar and the material is isotropic, whereas the latter is made of a complex 3D orthotropic structure that exhibits different mechanical properties along the three perpendicular directions of the wood (L, longitudinal; R, radial; T, tangential).

We varied the dimensions and the BCs of the aluminum rectangular plate to build an extensive dataset, whereas in the violin plate dataset, the outline of the plate was modified according to [43].

### 5.1. COMSOL Simulation

Simulations are based on the finite element method (FEM) [44] using *COMSOL Multiphysics*^®^ software to compute a numerical approximation of the sound pressure radiated and the velocity generated by the vibrating structure.

Both for the rectangular plate and for the violin top plate simulations, two steps have been applied. The first step involves a mechanical study in order to retrieve the eigenfrequencies $\bar{\omega }$ of each item in the dataset (*Eigenfrequency study*). In the second step, a suitable acoustic pressure study in the frequency domain has been accomplished (*Pressure Acoustics, Frequency Domain study*).

More specifically, in the acoustic simulation we emulated multiple shaker test setups by applying an external sinusoidal load at a fixed point on the structure with carrier frequency equal to each eigenfrequency computed in the previous mechanical study. Then, we retrieved the radiated sound pressure and the normal velocity associated to that specific vibrational input. In particular, we selected $\bar{\omega }\in \left[0,\omega_{\mathrm{MAX}}\right]$ where $\omega_{\mathrm{MAX}}$ is defined such that $\frac{\omega_{\mathrm{MAX}}}{2\pi }=2000\mathrm{Hz}$. Moreover, in order to validate the devised methodology in scenarios compatible to experimental NAH setups, we evaluated the holographic pressure at the elevation $z_{H}$ close to 3 cm.

In order to have accurate estimations of the complex fields, the discretization process consisted of second-order polynomial interpolation. In particular, the mesh elements were built in order to have at least five second-order elements for each wavelength, i.e., $h_{\mathrm{MAX}}=\lambda_{0}/5$, where $\lambda_{0}$ is the wavelength corresponding to the maximum frequency considered of 2000Hz.

Finally, we sampled the synthesized data with a cubic interpolation to yield the discrete estimations of $P_{H}\left(\omega \right)$ and V(ω) datasets. As far as V(ω) is concerned, it is sampled on rectangular grids with dimensions $L_{x}$ and $L_{y}$ in N=1024 points. The grid dimensions change accordingly to the specific object shape and size, such that the vibrating structure is entirely contained in the rectangular grid. Therefore, the sampling steps ${\bar{x}}_{s}$ and ${\bar{y}}_{s}$ of the normal velocity are computed according to

(13) $${\bar{x}}_{s}=\frac{L_{x}}{N_{2}-1},\;{\bar{y}}_{s}=\frac{L_{y}}{N_{1}-1},$$

with $N_{1}=16$ and $N_{2}=64$.

**Figure 4:**
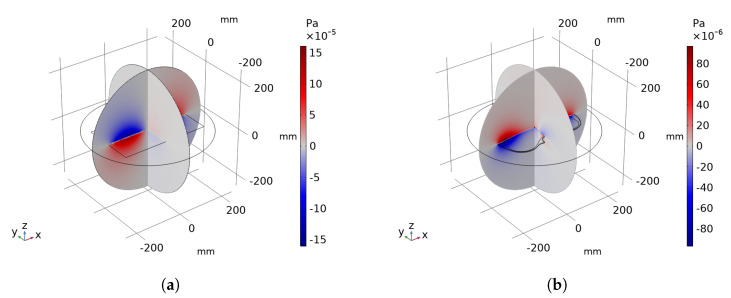
3D pressure radiation example of a rectangular plate (**a**) and a violin top plate (**b**) computed by the *Pressure Acoustics, Frequency Domain study* of *COMSOL Multiphysics*^®^.

As for the acoustic soundfield, an example of the 3D pressure radiation resulting from *COMSOL Multiphysics*^®^ is depicted in Figure 4. From the computed acoustic simulation, we retrieved $P_{H}\left(\omega \right)$ at the hologram plane H placed at $z_{H}$.

We sampled the hologram pressure in a uniform rectangular grid with the same dimensions $L_{x}$ and $L_{y}$ used for V(ω). We defined the pressure sampling steps in order to collect two different spatial resolutions version of $P_{H}\left(\omega \right)$ to validate the two proposed KHCNN architectures described in Section 4. In particular, we collected $P_{H}\left(\omega \right)$ in M=1024 points arranged in $M_{1}\times M_{2}=16\times 64$ and also in M=64 points arranged in a grid of 8×8.

Notice that, when $P_{H}\left(\omega \right)$ is sampled in M=1024 points, the input and output spatial resolutions are the same. Conversely, when $P_{H}\left(\omega \right)$ is sampled with M=64, we have fewer pressure points than velocity ones.

We modeled 672 different rectangular plates, with dimensions comparable to the body of small bowed-string instruments. In particular, with length $L_{x}\in \left[0.23,\;0.36\right]\;m$, width $L_{y}\in \left[0.15,\;0.22\right]\;m$, and thickness $L_{z}\in \left[0.002,\;0.007\right]\;m$. The $L_{x}$ and $L_{y}$ dimensions have been varied with step of 0.01 m while the $L_{z}$ dimension with 0.001 m.

For each plate, we analyzed with *COMSOL Multiphysics*^®^ the mechanical behavior for three different boundary conditions (BCs), i.e., simply supported, clamped, and free edges. To avoid exciting the plates on nodal lines and analyze as many different operational deflection shapes (ODS) as possible, we excited the plate with simply supported and clamped BCs at $x=L_{x}/5$ and $y=L_{y}/4$ locations, while for free BC, the excitation point was in the center of the plate.

We collected a dataset of 15,570 pairs of $P_{H}\left(\omega \right)$ and V(ω). In particular, we obtained 8707 instances for the free BC, while 2752 and 4111 correspond to clamped and simply supported BCs, respectively [45].

As for the violin plate dataset, we simulated 1568 different synthetic violin top plates with variable outline. Authors in [43] described 20 different parameters, which enable the complete definition of a violin top plate geometry, i.e., shape and dimensions. In [43], shape parameters are sampled from Gaussian distributions centered around the nominal value of a reference violin (based on a Stradivarius instrument). Thanks to this approach, we used different violin-like geometries with parametric outlines in order to ease a generalization on the 3D shapes. In *COMSOL Multiphysics*^®^ software, we modeled the radiated pressure and velocity data by exciting the center position of each violin top plate with free BC, which yields a total of 72,523 instances in the dataset.

### 5.2. Data Augmentation and Additive Noise

The pairs of $P_{H}\left(\omega \right)$ and V(ω) in the training set need to be the same for the simply supported, clamped and free BCs. This is especially true for the rectangular plate case, where we want the network to infer the different vibrational behaviors for the three BCs.

Nevertheless, the datasets resulting from the *COMSOL Multiphysics*^®^ simulation of rectangular plates are not well balanced. Due to the different dimensions considered, a larger number of items corresponding to modes at low frequencies can be found in the dataset with respect to the high frequency ones. Moreover, plates with clamped BC are characterized by modes with higher eigenfrequencies than free and simply supported ones.

For these reasons, we set up an analysis on the mode occurrences. Modes that are underrepresented in the dataset undergo a data augmentation step in order to have a balanced training set.

The mode occurrence analysis is based on the computation of a correlation matrix between all of the mode shapes present in the dataset. For all modes that are underrepresented, a replication is accomplished, so that a homogeneous distribution of the vibrational patterns is obtained.

Moreover, the collected $P_{H}\left(\omega \right)$ of violin and rectangular plates has been corrupted with additive white Gaussian noise in order to model the effect of measurement noise in the pressure sensors. This operation is accomplished for both 1024 and 64 sampled points data at the hologram.

The additive noise applied to each pressure item in the datasets is such that the signal-to-noise ratio (SNR) is selected from a uniform distribution in the interval [10,60] dB.

Table 1 reports the number *D* of $P_{H}\left(\omega \right)$ and V(ω) fields for the rectangular and violin datasets.

## 6. Validation and Results

In this section, we describe the experiments conducted and we discuss the related results.

### 6.1. Metrics

Two are the metrics used for assessing the performance of KHCNN. They both test the deviation of the estimated velocity field $\hat{V}\left(\omega \right)$ with respect to the ground truth V(ω) as computed from the *COMSOL Multiphysics*^®^ model.

For notational simplicity, in the rest of this section, we omit ω, but the dependence is implicit in the metric definition and in the complex field notations.

The normalized cross correlation (NCC) is a metric widely adopted in the NAH context that assesses the similarity between the prediction and the ground truth and it is defined as

(14) $$\mathrm{NCC}\left(\hat{v},\;v\right)=\frac{|{\hat{v}}^{H}\;\cdot \;v|}{{\left\|\hat{v}\right\|}_{2}\;\cdot \;{\left\|v\right\|}_{2}},$$

where the complex velocity fields are considered as column vectors. Notice that NCC is in [0,1] and it is equal to one if the two velocity fields match perfectly.

The second metric used to evaluate the accuracy is the normalized mean square error (NMSE), and it is defined in dB as

(15) $$\mathrm{NMSE}\left(\hat{v},v\right)=10\log_{10}\left(\frac{e^{H}\;\cdot \;e}{v^{H}\;\cdot \;v}\right),$$

where $e=\hat{v}-v$ is the column vector error between the prediction and the true value. It is worth noticing that NMSE emphasizes scaling and bias errors between $\hat{v}$ and v, which are not captured by NCC.

### 6.2. Validation

In this section, we evaluate the reconstruction capabilities of KHCNN with different boundary conditions (BCs). Moreover, we compare the results of the reconstruction when M=1024 or M=64 points are used on the holographic plane. For the ease of the reader, we will use the apex to emphasize the number of input points at the hologram pressure, e.g., $P_{H}^{\left(M=64\right)}$.

The test set consists of 1557 pressure fields of rectangular plates. In the following, the resulting NMSE and NCC are shown in octave band frequencies from the analysis of all the modes in the considered band. In particular, median values, standard deviation, and quartile distributions of NMSE and NCC in a band are depicted with box plots [46].

It is important to notice that, in the lower frequency bands, mainly free BCs are present, due to the different distribution of the eigenfrequencies along the frequency axis for the three BCs. In particular, a larger number of mode shapes occur at high frequencies. This produces a bias in the computation of the arithmetic mean of the metrics with respect the entire test set. For this reason, in order to correctly understand the overall network performance it is more insightful to consider the median value.

**Figure 5:**
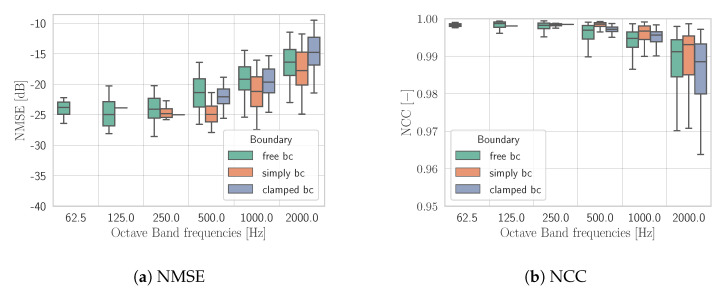
NMSE (**a**) and NCC (**b**) obtained by KHCNN adopting $P_{H}^{\left(M=64\right)}$. Results are shown in octave bands and grouped accordingly to BCs.

Figure 5 shows the metrics with M=64 points of hologram pressure subdivided for the three boundary conditions of the test set. By inspecting Figure 5, we can notice that a more accurate reconstruction is obtained for simply supported and clamped plates. A possible interpretation of this result can be found in the fact that with free BC, mode shapes are less predictable than with clamped and simply supported ones.

Nevertheless, KHCNN is able to recognize from the low spatial resolution input $P_{H}^{\left(M=64\right)}$ the different BCs applied to the vibrating source; thus, producing accurate $\hat{V}$ in N=1024 points. In particular, the estimates reached a NMSE<−10 dB for the whole test set and NCC steadily above 0.95.

**Figure 6:**
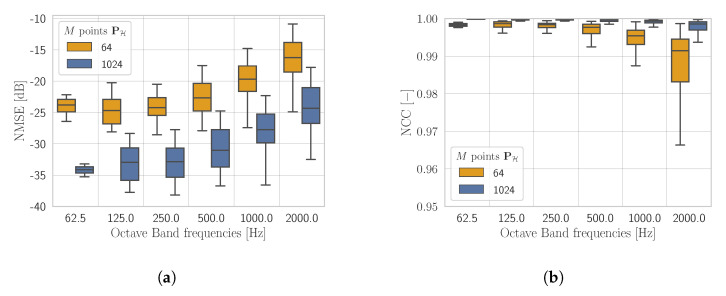
NMSE (**a**) and NCC (**b**) shown in octave bands given by the KHCNN estimates with $P_{H}^{\left(M=64\right)}$ and $P_{H}^{\left(M=1024\right)}$ at the input.

In Figure 6, we compare NMSE and NCC between the networks with $P_{H}^{\left(M=1024\right)}$ and $P_{H}^{\left(M=64\right)}$ at the input. Notice that, in this case, the plates in the test set have not been subdivided with respect to the BCs. From Figure 6 it is possible to observe that for both metrics the network based on an input of 1024 points offers an advantage with respect to the 64 points one, as one would expect.

It must be noticed that the KHCNN accuracy presents the same trend for M=1024 and M=64 for all frequency bands. In particular, the median NMSE value (Figure 6a) with the hologram pressure at M=1024 points is −27.49 dB and −19.65 dB for the case with M=64 input points. On the other side, the median value of NCC (Figure 6b) decreases from 99.92% for M=1024 points to 99.53% for the M=64 case. These results highlight scaling differences between the reconstructions produced in the two cases. Nevertheless, by looking at the definition of NMSE (15), the decrease due to lower input dimensionality corresponds to a relative error of the two median values around 16%.

**Figure 7:**
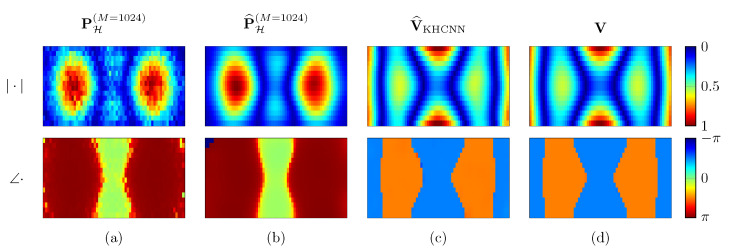
KHCNN reconstruction example for a rectangular plate with dimensions $\left[L_{x},L_{y},L_{z}\right]=\left[0.33,0.15,0.002\right]m$ vibrating at 548Hz. The first and the second row depict the magnitude and phase of the complex fields, respectively. Column (**a**) is the input pressure at the hologram in M=1024 points. The reconstructed pressure from the KH model is shown in column (**b**). The velocity estimate and ground truth are depicted in column (**c**,**d**), respectively.

Figure 7 shows the KHCNN reconstruction of a rectangular plate vibrating at 548Hz with free BC. Although the network input $P_{H}^{\left(M=1024\right)}$ is corrupted with additive white Gaussian noise (SNR=13.8 dB), the velocity estimate ${\hat{V}}_{\mathrm{KHCNN}}$ reaches a NCC value of 99.85% and NMSE=−24.97 dB.

Moreover, a comparison between the reconstructed hologram pressure ${\hat{P}}_{H}^{\left(M=1024\right)}$ computed with the KH model using ${\hat{V}}_{\mathrm{KHCNN}}$ and the input pressure $P_{H}^{\left(M=1024\right)}$ is given in Figure 7. We can notice that ${\hat{P}}_{H}^{\left(M=1024\right)}$ presents smoother patterns both for the magnitude and for the phase rather than the input $P_{H}^{\left(M=1024\right)}$. Therefore, KHCNN is able to perform a denoising operation at the hologram pressure. This is a general behavior obtained for all KHCNN reconstructions of the test set and it is more visible for the estimates that start with low SNR values of input pressures.

### 6.3. Comparison with the Baseline

To validate the devised methodology with respect to state-of-the-art approaches, we compared the estimations obtained by KHCNN with a model-based and a fully data-driven approach adopted in the context of NAH. In particular, we compared the rectangular plate reconstructions computed with the devised KHCNN and the CESM presented in [20]. Furthermore, the velocity reconstructions on the violin top plates was compared with the SRCNN-NAH approach presented in [31].

Similarly to [20], here, we solved the optimization problem of CESM with the CVX solver [47]. The equivalent point sources are distributed on a uniform planar grid of 25×25 points located at the height $z_{eq}=-5\;cm$ behind the surface of the plate. Moreover, the grid of equivalent point sources is 1 cm larger, with respect to each edge of the rectangular plate under study.

**Figure 8:**
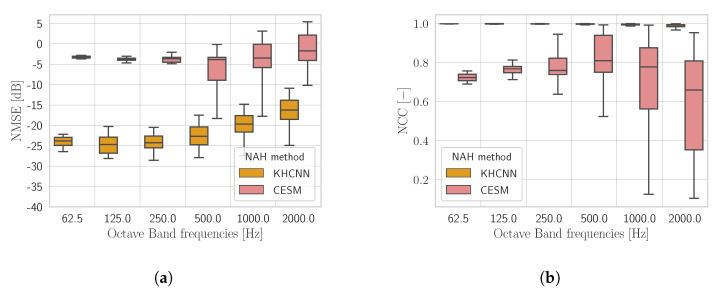
The NMSE (**a**) and the NCC (**b**) comparison between the rectangular plate estimates of KHCNN and CESM from $P_{H}^{\left(M=64\right)}$.

Figure 8 shows the comparison between KHCNN and CESM for what concerns the velocity field reconstruction of the rectangular plate test set starting from $P_{H}^{\left(M=64\right)}$.

In general, the devised KHCNN outperformed the CESM approach in the whole frequency range both for NMSE and for NCC. In particular, the median NCC value for the KHCNN reconstructions is 99.53%, while for CESM it is equal to 76.58%. Moreover, the NMSE median value decrease from −19.65 dB for KHCNN to −3.46 dB for CESM.

By inspecting the reconstructions, we noticed that KHCNN is more robust than CESM to the presence of noise in the input pressure. Moreover, although CESM obtains good accuracy for the simply and clamped BCs, the overall trend decreases due to the reconstructions with free BC. This is confirmed by the median NCC values of CESM that correspond to 89.8%, 79%, and 74.3% for the simply, clamped, and free BCs, respectively.

**Figure 9:**
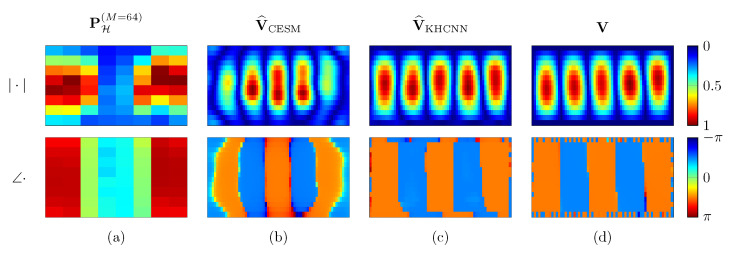
Reconstruction example of CESM and KHCNN for a simply supported rectangular plate with dimension $\left[L_{x},L_{y},L_{z}\right]=\left[0.32,0.17,0.002\right]m$ vibrating at 1371Hz. The first and the second row depict magnitude and phase of the complex fields, respectively. Column (**a**) is the input pressure at the hologram in M=64 points. The CESM and KHCNN velocity estimate are depicted in column (**b**) and (**c**), respectively. Column (**d**) shows the velocity ground truth coming from simulation.

An example of KHCNN and CESM estimate is shown in Figure 9. Both techniques reconstructed the surface normal velocity of the rectangular plate with simply supported BC at 1371Hz from $P_{H}^{\left(M=64\right)}$. Nevertheless, the ${\hat{V}}_{\mathrm{KHCNN}}$ is more accurate than the ${\hat{V}}_{\mathrm{CESM}}$ both for the magnitude and for the phase. This is confirmed also by the metrics. In particular, the NCC values for CESM and KHCNN are 87.39% and 99.56%, respectively. This is also confirmed by the fact that NMSE=−6.19 dB for the CESM estimate, while with KHCNN, we obtain NMSE=−20.47 dB.

Furthermore, in order to validate the proposed KHCNN to objects different from rectangular plates, we analyzed the velocity reconstructions on violin top plates. Moreover, we compared the results of KHCNN with the ones obtained with the SRCNN architecture proposed in [31]. Hence, we trained the two systems with $P_{H}^{\left(M=64\right)}$ and V pairs of violin top plates generated with COMSOLMultiphysics® software.

**Figure 10:**
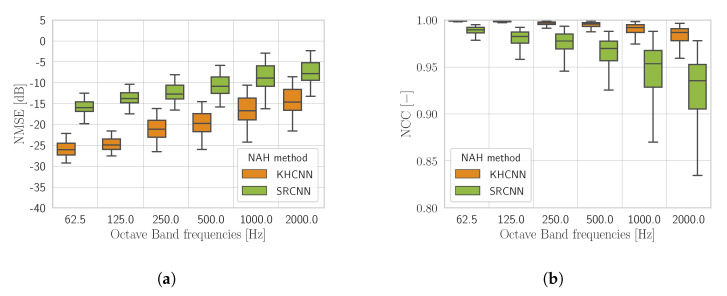
The NMSE (**a**) and the NCC (**b**) comparison between the violin top plate estimates of KHCNN and SRCNN with $P_{H}^{\left(M=64\right)}$ at the input.

Figure 10 shows the performance comparison of NMSE and NCC for 7266 pressure fields of violin top plates. Notice that, since SRCNN reconstructs only the magnitude of the velocity field, the metrics are computed, considering the absolute value of the velocity estimates coming from KHCNN.

In general, NMSE and NCC for both architectures present the same trend, with a more accurate reconstruction in the lower frequency bands with respect to higher ones. Nevertheless, KHCNN can achieve higher accuracy with a NMSE median value of −17.10 dB and a median NCC of 99.24%. The reconstructions with SRCNN, instead, reached a NMSE and NCC median value of −9.31 dB and 95.58%, respectively.

**Figure 11:**
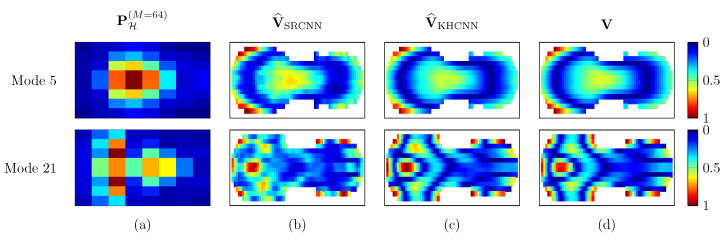
Reconstruction examples of SRCNN and KHCNN for two different violin top plates. The first and second row show the magnitude vibration of mode 5 and 21 at 264Hz and 948Hz, respectively. Column (**a**) is the input pressure at the hologram in M=64 points. The SRCNN and KHCNN velocity estimates are depicted in column (**b**,**c**), respectively. Column (**d**) shows the magnitude velocity ground truth coming from simulations.

Two examples of the velocity magnitude estimates can be seen in Figure 11. The devised architecture is able to obtain a more accurate and smoother velocity pattern than the SRCNN one. As a matter of fact, in the second reconstruction example (at 948Hz), the NMSE value of SRCNN and KHCNN are −9.17 dB and −16.07 dB, respectively. Likewise, NCC=94.19% for SRCNN and for KHCNN the NCC value is 98.82%.

**Figure 12:**
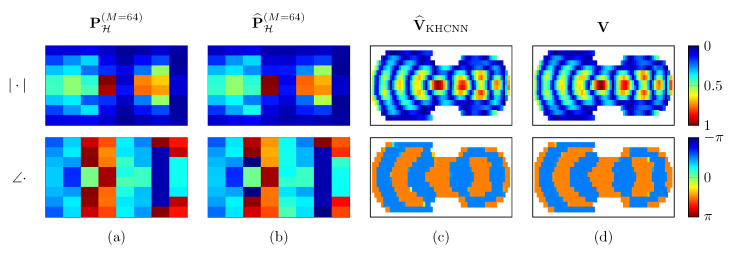
Reconstruction example of KHCNN for a violin top plate vibrating at 1829Hz. The magnitude and phase of the complex fields are represented in the first and second row, respectively. Column (**a**) is the input pressure at the hologram in M=64 points. The hologram pressure computed with the KH propagation is shown in column (**b**). The velocity estimate and ground truth are depicted in column (**c**,**d**), respectively.

Moreover, in addition to the more accurate results for the magnitude reconstructions, KHCNN is able to estimate the phase information of the desired V. In Figure 12 an example of the KHCNN reconstruction, i.e., complex velocity and hologram pressure fields, for a violin top plate vibrating at 1829Hz is shown.

## 7. Discussion

From the analysis of the results displayed in the previous section, KHCNN shows accurate reconstructions with different shapes and mechanical properties of vibrating sources, improving the performance with respect to sparsity-based NAH [20] and recent DNN [31] solutions.

Interestingly, KHCNN is able to retain accurate estimates when the size of the input data is reduced to one-sixteenth of the output spatial resolution. Hence, KHCNN effectively achieves the super-resolution of velocity fields as in [31], while improving the overall accuracy of the reconstruction. In particular, KHCNN with M=64 input pressure points of a rectangular test set produces the complex velocity field in N=1024 points with a median NMSE value stable around −25 dB for the first four octave bands, i.e., up to 707Hz.

With respect to the CESM approach [20], KHCNN shows improved results, in terms of NMSE and NCC. As a matter of fact, KHCNN is able to produce more accurate estimates of the complex velocity field, i.e., considering both magnitude and phase. The accuracy of CESM greatly degrades when free BCs are adopted. KHCNN, instead, is able to limit the performance reduction obtaining comparable results for all three BCs under analysis. It is worth noticing that the CESM approach is led by an approximation of the acoustic propagation model. The velocity field, indeed, is estimated from a sparse set of point-like sources, which equivalently describe the soundfield at the holographic plane. Conversely, KHCNN employs a neural network to estimate the velocity field and it takes advantage by the actual propagation model represented by the Kirchhoff−Helmholtz equation.

Results show that the desired velocity field can be computed on the surface of complex shapes that present arching, such that violin top plates. Here, KHCNN is compared with respect to SRCNN [31], a recently developed DNN-based NAH technique. The improved results attained using KHCNN can be noticed especially in the lower frequency bands, where the difference between the statistical distribution of the metrics is substantial. In particular, KHCNN reduces the NMSE values of around 10 dB with respect to SRCNN up to 353Hz. In the highest frequency range, the difference between KHCNN and SRCNN is not significantly reduced.

The main difference between the two architectures lies in the propagation model introduced in KHCNN. As a matter of fact, SRCNN learns a direct mapping between the magnitude of the input pressure and the magnitude of the surface velocity. Although effective, this approach does not take into account for possible deviations in the soundfield generated by the estimated velocity. Differently from the end-to-end approach of SRCNN, KHCNN explicitly considers the complex velocity field (real and imaginary parts) and the propagation through the Kirchhoff−Helmholtz equation. As a result, the estimated velocity fields improved considerably, allowing us to obtain a more accurate reconstruction of the violin mode shapes.

Lastly, we can observe that KHCNN performed consistently on both the datasets under analysis despite the different shapes, BCs and material properties of the objects. As a matter of fact, in the case of rectangular plates with 64 points at the input the median values of NMSE and NCC are −19.65 dB and 99.53%, respectively. Similarly, for the violin plate dataset, we obtained a median NMSE value of −16.83 dB and a NCC median of 99.2%, both computed with the complex fields. Hence, although the violin plates present diverse shapes and material properties, the difference on the median values is limited to 2.82 dB and 0.33% for the NMSE and NCC, respectively. The proposed CNN approach aims, therefore, to provide a promising tool for a wide variety of NAH applications.

## 8. Conclusions

In this manuscript, we introduced a novel technique for NAH. The devised architecture, called KHCNN, combines the advantages of the learning feature of CNN with the physical information given by the Kirchhoff−Helmholtz forward propagation model. In particular, the CNN is trained in order to provide an estimate of the velocity field of the source starting from the acquired acoustic pressure. Through the propagation of the estimated velocity using the Kirchhoff−Helmholtz equation, the prediction was then refined, comparing the respective acoustic pressure with the input data.

The proposed KHCNN was validated with two different datasets: isotropic rectangular plates and orthotropic violin top plates. The velocity ground truth on the vibrating structures and the complex pressure field at the holographic plane were generated for each structure using the finite element method with *COMSOL Multiphysics*^®^ software. We varied the dimensions and the boundary conditions for each vibrating plate to ease a generalization of the method. Moreover, the synthesized pressures were corrupted with different SNRs of additive white noise in order to simulate sensor noise.

Results show that KHCNN is able to estimate the desired complex velocity field on vibrating objects, starting from the low spatial resolution of radiated soundfield. We obtained accurate reconstructions for both the magnitude and the phase information of the vibrational field. In particular, KHCNN reached a median NMSE value under −16 dB and a median value of NCC above 99% for both the rectangular plate dataset and the violin plate one. Moreover, the explicit definition of the forward propagation model into KHCNN enables further verification of the network estimates by comparing the pressure reconstruction at the hologram.

Furthermore, we assessed the network accuracy with respect to recent NAH approaches available in the literature. We compared the rectangular plate estimates of KHCNN with CESM and the magnitude of violin top plate reconstructions with the fully data-driven approach of SRCNN. In both cases, the KHCNN results outperformed the considered approaches in terms of normalized mean square error and normalized cross correlation for the whole frequency range considered.

Future works will be devoted to the application of KHCNN to experimental measurements following two main directions. On one side, we aim at training the architecture using simulations while testing the system on the field on real data acquired also in the presence of reverberation. This will allow us to avoid expensive and time consuming measurement campaigns exploiting the flexibility of simulations for building extensive datasets with variable characteristics offline. On the other side, we expect to extend the KHCNN approach to work with a wide variety of objects without explicitly retraining the network. For both points, we foresee the application of domain adaptation and transfer learning strategies in order to tune the network with different data.

## Figures and Tables

**Table 1:** Dimension of the datasets.

Dataset	Total Dimension
Rectangular plate	$D_{rectangular}$ = 186,294
Violin top plate	$D_{violin}$ = 72,523

## Data Availability

The rectangular plate dataset simulated with *COMSOL Multiphysics*^®^ software are openly available in Zenodo at [45], reference number 10.5281/zenodo.5702615. Moreover, the implementation of KHCNN and the trained weights are available in the GitHub repo (https://github.com/polimi-ispl/nah-khcnn, accessed on 20 November 2021).

## Abbreviations

The following abbreviations are used in this manuscript:

NAH	nearfield acoustic holography
DNN	deep neural network
CNN	convolutional neural network
KH	Kirchhoff−Helmholtz
BC	boundary condition
KHCNN	Kirchhoff−Helmholtz-based convolutional neural network
CESM	compressive equivalent source method
SRCNN	super resolution convolutional neural network
